# The immunogenicity of colorectal cancers with high-degree microsatellite instability

**DOI:** 10.1186/1477-7819-3-26

**Published:** 2005-05-12

**Authors:** Ayan Banerjea, Stephen A Bustin, Sina Dorudi

**Affiliations:** 1 Centre for Academic Surgery, Barts and the London Queen Mary School of Medicine and Dentistry, London, UK

## Abstract

**Background:**

High-degree microsatellite instability (MSI-H) is a feature of approximately 15% of sporadic colorectal cancers. Patients with MSI-H cancers have been reported to have a better prognosis than those with non-MSI-H cancers. The MSI-H subset is also characterised by a dense infiltrate of intra-epithelial lymphocytes and the hypothesis that the latter represents an efficacious immune response contributing to improved outcome is very attractive.

**Methods:**

Data for this review were identified by searches of MEDLINE, PubMed, and cross references from relevant articles using the search terms 'microsatellite instability', 'colorectal cancer' and 'immunology', 'immune response' or 'immunogenicity'.

**Results:**

A total of 38 articles were identified by the search criteria and a further 95 articles by cross-referencing. The relevance of the articles to be interviewed was established by hand searching. Out of a total of 133 articles identified, 47 articles were rejected due to lack of relevance. A total of 86 articles were included in the review, pertaining to microsatellite instability in colorectal cancer, and immune mechanisms in colorectal cancer.

**Conclusion:**

It is suggested that this distinct group of colorectal cancers may have inherent immunogenic properties and that further elucidation of these may be invaluable to the development of successful immunotherapy.

## Background

Colorectal cancer remains a leading cause of cancer-related mortality and morbidity in the Western world. In the UK it is the third commonest cancer, after lung and breast, with more than 35,000 new cases diagnosed each year and the second commonest cause of cancer-related mortality with approximately 16,000 deaths per year (CancerStats: Large Bowel – UK, Cancer Research UK 2003, [1]). Complete surgical resection is the cornerstone of curative treatment. Other treatment modalities, such as chemotherapy and radiotherapy, administered before or after surgery, are used to counteract disease progression particularly in Stage III and IV disease [2,3]. Despite continuing technical advances in the use of these techniques the improvement in overall five-year survival rate has been limited (5–6%). This fact, along with the high proportion of patients who present with tumours not amenable to surgery, drives the search for additional modalities of anti-cancer treatment. Immunotherapy, the use of the adoptive immune system to target cancer cells, holds much promise in this regard.

The concept of immune surveillance, whereby the host immune system detects and removes cancerous cells, arose from the observation that immunodeficiency predisposes to the development of malignancies. Furthermore, it has been proposed that rare reports of solid tumour regression are the result of an anti-tumour immune response. Early work suggested that such anti-tumour responses were impotent against colorectal cancer [4-6]. However, evidence that colorectal malignancies may generate tumour specific antigens (TSA's) has encouraged considerable efforts to develop means of harnessing the host immune system to combat colorectal cancer. The use of immunotherapeutic approaches in colorectal cancer has evolved substantially (not reviewed here) and now focuses on the use of tumour specific antigens. This involves either passive immune therapy with antibodies targeted directly to tumour cells or by active immune therapy via vaccination with tumour cells, tumour cell lysates, peptides, carbohydrates, gene constructs encoding proteins, or anti-idiotypic antibodies that mimic TSA's. Despite some successes these techniques are still in their infancy and many hurdles remain, largely because our understanding of the biology of host-tumour interactions in colorectal cancer remains elementary.

In the last ten years a distinct subset of colorectal cancer, with characteristics of widespread microsatellite instability, has emerged as a group in which immune responses may be pronounced. Clearly, if this type of colorectal cancer does indeed have particular immunogenic features, its study should provide vital advances in our understanding of colorectal tumour immunology. Here we review the literature in relation to putative immune responses in colorectal cancers with microsatellite instability.

### Microsatellite instability (MSI)

It is now clear that colorectal cancers may arise by a number of distinct molecular pathways. The "classical pathway" characterised by chromosomal instability (CSI) and a series of genetic events that drives the progression from normal colon through adenomatous polyp to invasive cancer accounts for 50–60% of cancers [7]. However, this is but one of many alternate pathways and one of these, involving mismatch repair dysfunction, leads to colorectal cancer characterised by microsatellite instability. High-degree microsatellite instability (MSI-H) is a feature of most colorectal cancers arising as part of the hereditary non-polyposis colorectal cancer (HNPCC) syndrome and also 15–20% of sporadic colorectal cancers [8].

Microsatellites are short segments of repetitive DNA bases found throughout the genome but mainly located in intronic (non-coding) segments. Due to their repetitive nature they are prone to insertion and deletion mutations during DNA replication, which commonly introduces such errors at a rate of 1 in 100,000 base pairs copied. The majority (99%) of these errors are detected and corrected by the inherent proof reading capabilities of DNA polymerase enzymes. A small proportion of mismatches that are missed by this mechanism are normally identified and corrected by a group of proteins collectively termed the mismatch repair (MMR) system [9]. Dysfunction of the MMR system allows the accumulation of replication errors and leads to microsatellite instability [10], defined as "a change of any length due to either insertion or deletion mutations of repeating units in a microsatellite" [11].

### Mismatch repair dysfunction

The molecular mechanisms that underlie MMR dysfunction are different in familial and sporadic colorectal cancers. In HNPCC colorectal cancers a germline mutation within an individual mismatch repair gene is inherited and a subsequent "second hit" [Knudson's 'two-hit' hypothesis [12]] in the normal allele leads to mismatch repair deficiency. The commonly affected genes are hMLH1, hMSH2 and to a lesser extent PMS2, and their involvement predicts the development of extensive microsatellite instability [13]. By contrast, cancer cells with isolated mutations in hMSH3 and hMSH6 continue to maintain some mismatch repair function [14-16].

In sporadic MSI colorectal cancers genetic alterations in MMR genes are relatively rare. Instead epigenetic modulation of MMR genes, particularly hMLH1, such as hypermethylation of the promoter region leads to silencing and MMR deficiency [17,18]. Hypermethylation is characteristic of a subset of colorectal cancers designated the CpG island methylator phenotype (CIMP+) that is relatively common in right sided tumours [19]. Sporadic MSI-H colorectal cancer appears to represent a subset of the methylator phenotype in which global hypermethylation silences MMR genes. These cancers have biological properties quite distinct from that of HNPCC tumours, in which MMR dysfunction is the sole abnormality, whereas widespread changes in methylation targets the expression of numerous other genes not involved in the MMR pathway [20].

All colorectal cancers display some degree of microsatellite instability if enough markers are studied [21]. In 1997 an International Collaborative Group laid down guidelines for the identification and assessment of microsatellite instability [22]. They classified tumours into microsatellite stable (MSS) and two groups of microsatellite unstable cancers, discriminated by their degree of instability: high (MSI-H) or low (MSI-L). Two mononucleotide microsatellite markers (BAT-25 and BAT-26) and three dinucleotide markers (D2S123, D5S346 and D17S250) were chosen as a panel to be studied for assignation of MSI status. Tumours displaying instability in two or more markers were designated MSI-H, instability in only one marker was designated MSI-L and if no markers were affected the tumour was designated MSS. There is now a growing consensus that the MSI-H clinico-pathological phenotype is characterised particularly by instability in the mononucleotide markers [21]. Colorectal cancers with MSI-H clearly demonstrate distinct biological behaviour whereas MSI-L cancers are less well characterised and are often grouped with cancers that show no instability (MSS) [23]. The focus of this review is the clinico-pathological phenotype characteristic of sporadic MSI-H colorectal cancer.

#### MSI-H colorectal cancer

Sporadic MSI cancers do not harbour some of the characteristic features or gene mutations associated with CSI cancers, such as aneuploidy and *p53 *mutation. A number of human genes implicated in pathways regulating growth control and apoptosis, for example *IGF-IIR, TGFβ-RII *and *BAX*, contain microsatellite repeats within the coding regions of their transcribed sequences. More recently the *BRAF *gene has been noted to be commonly mutated in sporadic MSI-H, as well as in serrated polyps, suggesting that serrated polyps are likely precursors of these cancers [20]. It has been suggested that instability of such sequences plays a role in the progression of disease, presumably by altering gene activity [8,24]. Altered regulation of all or some of these genes may be involved in cancer development but precise mechanisms are yet to be identified.

Colorectal cancers with MSI-H also have distinct clinico-pathological characteristics. Sporadic MSI-H tumours are associated with the right side of the colon and display marked lymphocyte infiltration into tumour epithelium, stroma, peri-tumoral cuffs and Crohn's type reactions [25]. Sporadic MSI-H colorectal cancers have also specifically been associated with medullary-type poor differentiation, high mucin content and reduced lymph node involvement [20,25].

#### MSI-H and improved survival

Early reports on microsatellite instability noted an association of MSI with improved patient survival [26,27]. Some small studies have failed to reproduce this finding [28-30] but generally the association of MSI-H with improved survival in sporadic cancers has been confirmed in larger studies [31-33]. Many further studies have followed that confirm improved prognosis for patients with sporadic MSI-H colorectal cancer, particularly in lymph node positive cancers, and the link to improved survival has now been confirmed in a systematic review [34].

One explanation offered for the improved survival of MSI-H colorectal cancer patients has been that these cancers may be more sensitive to adjuvant chemotherapy. Indeed early retrospective analyses suggested that cancers with high-degree microsatellite instability had improved response to 5-Fluorouracil (5FU)-based adjuvant chemotherapy [35-37]. However, these data were retrospectively derived from non-randomised groups and the effects of small numbers, younger age of patients receiving chemotherapy, and different responses of right and left-sided tumours might have confounded the findings. In contrast, two studies have reporting retrospective data derived from randomised control trials of chemotherapy conclude that 5-FU regimes confer no benefit in sporadic MSI-H colorectal cancers and may even be detrimental [38,39]. There is clearly a need for robust prospective randomised controlled trials to evaluate the effects of 5-FU, and other agents, on survival with respect to MSI status.

An alternative explanation for the improvement in patient survival is one of enhanced host immune response. An immunohistochemical study noted that the improved survival of patients with MSI-H was associated with the higher frequency of activated tumour infiltrating lymphocytes in these cancers [40]. Consequently, it has been suggested that these lymphocytes may actually represent a host immune response that contributes to improved survival and subsequent work has confirmed a possible link [41]. The hypothesis that colorectal cancers with MSI-H are more immunogenic than microsatellite stable cancers is very attractive, as study of these cancers may provide crucial insights into colorectal cancer immunology. The further elucidation of immune responses to colorectal cancer *in vivo *is crucial to the continuing development of immunotherapy.

#### MSI-H and tumour immunogenicity

Current concepts in solid tumour immunology identify certain features that are fundamental to the stimulation of an anti-tumour response [Reviewed in [42]]. Successful immune response requires a cancer to possess antigens that can be recognised as non-self during immune surveillance. In MSI-H colorectal cancer the accumulation of errors in microsatellites, that reside in gene exons, leads to the generation of a large number of abnormal peptides due to frameshift mutations: the so-called "mutator phenotype" [43]. Clearly, such an array of abnormal peptides might represent a pool of TSA's that render MSI-H tumours inherently more detectable by the host immune system.

Studies on abnormal peptides characteristic of MSI-H have demonstrated their potential for immune recognition. A peptide generated by frameshift mutations in TGFβRII, characteristic of the mutator phenotype, has been used to pulse dendritic cells, which in turn induced activated cytotoxic (CD8+) T cells [44]. One cytotoxic T cell clone demonstrated specific anti-tumour activity against a cell line expressing this peptide in *in vitro *conditions. The same group have also identified MHC class-II restricted epitopes by induction of helper (CD4+) T cells from the peripheral blood mononuclear cells of normal volunteers and MSI-H colorectal cancer patients [45]. They also demonstrated that tumour infiltrating lymphocytes derived from an HNPCC MSI-H cancer recognised this frameshift-mutation-derived peptide but unusually the infiltrate in this tumour was predominantly CD4+ T cells. More recently, another group have identified several other frameshift-mutation-derived peptides as potential tumour specific antigens (TSA's) by using SEREX (serological analysis of recombinant cDNA expression cloning) [46]. They also detected IgG antibodies specific to these peptides only in the MSI-H cancer patients. In an HNPCC patient they demonstrated an antibody specific to a mutated CDX2 peptide that was found in the tumour tissue of the patient and this antibody was not seen in any of the other patients. Interestingly, the antibody disappeared seven years after curative resection. These observations lend significant support to the notion that MSI-H TSA's are effectively recognised by the host immune response and an immune response is induced, but more work is needed to confirm that findings in HNPCC patients are mirrored in sporadic MSI-H cancers.

Tumour specific antigens must be appropriately presented to the host immune system if a specific immune response is to occur. This normally requires the presentation of antigen in conjunction with the appropriate Major Histocompatibility Complex (MHC) molecules that interact with the T cell receptor (TCR): Class I MHC for presentation to cytotoxic (CD8+) T cells and Class II MHC for presentation to helper (CD4+) T cells [47]. It has been argued that MSI-H colorectal cancers are incapable of eliciting cytotoxic responses *in vivo *due to the truncation of β_2_-microglobulin that is characteristic of these tumours [48]. The β_2_-microglobulin protein is an essential co-factor for MHC Class I and its defective function would render effective presentation of antigens impossible. However, whilst this defect negates antigen presentation by the tumour itself there is now considerable evidence that the direct presentation of antigen by a tumour may be relatively unimportant. Instead, the concept of "cross-priming" in which APC's such as dendritic cells, and to a lesser extent macrophages, pick up antigens released by dead tumour cells and subsequently present them to T cells may be more important *in vivo *[49,50]. Thus, β_2_-microglobulin mutations may be of relatively little importance. Instead a pool of abnormal peptides generated by the mutator phenotype may be recognised and harvested as TSA's by immature dendritic cells. On acquisition of non-self antigens these dendritic cells mature into professional APC's, with a natural propensity to appropriately present antigen and co-stimulate T lymphocytes [50].

Antigen presenting cells may obviate the need for direct presentation by tumour cells and cross-priming may involve the use of HLA Class II machinery. Immunohistochemical studies have demonstrated that HLA Class II expression is increased in MSI-H colorectal cancer compared to MSS cancers, although this was not associated with improved survival [51]. Additionally, molecular analyses using single subtraction hybridisation [52] and oligonucleotide microarrays [53] have also confirmed increased Class II mRNA levels in MSI-H colorectal cancer. These findings support the *in vitro *studies of tumour infiltrating lymphocytes in MSI-H colorectal cancer, mentioned earlier, that demonstrated HLA Class II restricted T helper cell activity and the presence of dendritic cells [44,45]. Either of these two populations may provide an alternative pathway by which cytotoxic T cells may be recruited and stimulated indirectly, but more work is needed to ascertain whether cytotoxic populations themselves are capable of recognising TSA's.

It has recently become evident that appropriate co-stimulation is pivotal to effective antigen presentation [54]. Co-stimulation refers to the interaction between surface molecules on the antigen-presenting cell (APC), such as CD80 (B7.1) and CD86 (B7.2), and surface molecules on the T cells (CTLA-4, OX40, CD40-L). If these molecules do not bind each other during antigen-presentation then co-stimulation fails and the T cells become anergic, i.e. ineffective and tolerogenic [54]. A microarray analysis comparing the gene expression profiles of MSI-H colorectal cancers to MSS counterparts demonstrated increased signal intensity of CD80 and CD86 in the former group [53] but further studies to validate these findings are still required. After appropriate antigen-presentation and co-stimulation lymphocytes are activated in a tumour-specific response [49,54]. They may then migrate to the tumour, infiltrate it and release mediators that can induce tumour cell death [47].

#### MSI-H colorectal cancer and lymphocyte infiltration

For many years the presence of a pronounced infiltrate of lymphocytes in colorectal cancer has been associated with improved prognosis [55,56] but conclusions have been confounded by inconsistencies. Lymphocytes associated with colorectal cancer can be sub-divided into those that invade tumour epithelium (intra-epithelial lymphocytes, IEL's), those confined to the stroma or those aggregated around the tumour (peri-tumoral lymphocytes). Previous studies on the significance of tumour infiltrating lymphocytes have been confusing due to variability in the definition of the subset being studied. More recently it has been shown that the presence of IEL's is positively associated with an improved survival in colorectal cancer in general [57] and specifically MSI-H colorectal cancer [41]. It has been suggested that they IEL's may represent evidence of an immune response to tumour antigens.

In a study specific to MSI-H colorectal cancer, Dolcetti and colleagues demonstrated increased immunostaining for IEL's when compared to MSS cancers [58]. These lymphocytes were shown to be predominantly CD8+ in keeping with a cytotoxic lymphocytic response and these findings have been confirmed by other groups using immunohistochemistry and RT-PCR [41,59]. However, debate continues as to whether these lymphocytes are of the αβT cell receptor lineage that are involved in tumour infiltration from the circulation or whether they are derived from γδ T cells that normally reside in the gastrointestinal tract [60]. The αβT cell phenotype represents lymphocytes that are targeting the tumour after antigen priming in lymphoid tissue, whereas the γδ T cells might represent a proliferation of lymphocytes resident in tumour epithelium. These characteristics of the lymphocyte infiltrates need further clarification.

One immunohistochemical study, involving relatively small numbers and two groups that differed considerably in size (n = 17 v n = 7), has noted the increased presence of CD8+ CD103+ (α^E^β_7_+) lymphocytes in MSI-H colorectal cancer compared to MSS cancers [61]. The presence of CD103+ cells was much higher in tumour tissue than in adjacent colon in both groups and the authors' postulate that this integrin subunit, which binds to E-Cadherin, has a role in migration of lymphocytes from stroma to tumour epithelium. They suggest that the differences in lymphocyte infiltration between the two groups are likely to be due to increased expression of CD103 by local lymphocytes in MSI-H cancer that promotes infiltration into the tumour. They also postulate that local mediators released by the tumour, such as TGFβ-I rather than a systemic immune response, leads to CD103 upregulation, but they provide no evidence to support this. The expression of CD103 may well facilitate lymphocyte infiltration from stroma to tumour, but whether the difference in IEL numbers between MSI-H and MSS is due to tumour-derived mediators or an immune response is unclear from this study [61].

The increased expression of cytolytic mediators by IEL's in MSI-H colorectal cancer suggests that these cytolytic T-cells are indeed activated. Increased expression of perforin, granzyme B and granulysin in MSI-H colorectal cancers [53,59,62] suggests that IEL infiltrates are attempting to induce tumour cell death. This is lent further credence by the finding that the activation marker IL2Rα is also expressed more significantly in these tumours [59]. No single activation marker has been shown to correlate with survival in studies to date but this may well due to the complexities of immune responses.

The process of lymphocyte infiltration and anti-tumour activity is also influenced by the differential activity of helper (CD4+) T lymphocyte subsets and local cytokine profiles. Immune responses are promoted by the Th1 subset of CD4+ lymphocytes and their associated cytokines [Interferon (IFN)-γ, Tumour necrosis factor (TNF)-α, Interleukin (IL)-2 and IL-12). By contrast the Th2 subset of CD4+ lymphocytes and cytokines such as IL-10 promote tolerance to unrecognised antigens and prevent successful antigen-specific responses. A microarray analysis performed in our laboratory has demonstrated the upregulation of several pro-inflammatory cytokines in MSI-H colorectal cancer [53]. As well as the common players this analysis has identified potentially crucial roles for IL-18 and IL-15, both of which are capable of co-ordinating innate and acquired responses [63,64]. Clearly, activity of these interleukins should promote the likelihood of a successful immune response.

This microarray analysis also demonstrated that the heat shock protein family is up-regulated in MSI-H colorectal cancer [53]. This is highly significant in the context of previous work by Srivastava and others that has demonstrated the role of heat shock proteins in innate and antigen-specific immune responses [65,66]. Heat shock proteins act as chaperones that are actively involved in regulating the folding of newly synthesised proteins [67]. Indeed their name derives from the observation that these proteins are over-expressed in response to heat shock, and other stresses, that lead to native protein unfolding. The native fold of a protein is encoded by its amino acid sequence and it is therefore attractive to suggest that cancers with high-degree microsatellite instability, that generate large numbers of abnormal proteins with consequent misfolding, might excite a heat shock protein response. Heat shock proteins are used as natural adjuvants in immunotherapy trials and their up-regulation in MSI-H tumours may reflect a similar function in naturally occurring immune responses [68,69].

Our microarray findings have been validated using quantitative real-time RT-PCR across several genes of interest and demonstrate clear differences in the tumour biology of MSI-H and MSS cancers at the mRNA level.

### Tumour escape and counter-attack

To counteract immune surveillance solid cancers may employ mechanisms to evade the immune system. Several possible methods of immune escape have been identified in colorectal cancer, such as the down-regulation of MHC proteins and co-stimulatory molecules [70,71]. The available evidence suggests that MSI-H colorectal cancers demonstrate higher levels of MHC Class II machinery than MSS counterparts, although the inefficacy of HLA Class I molecules in MSI-H tumours has been proposed as a means of tumour escape [48]. The significance of this in light of the potential for immune cross-priming is controversial, as discussed earlier.

Alternative mechanisms of immune escape identified in colorectal cancer include the release of mediators that suppress the function of tumour infiltrating lymphocytes: so-called "tumour counter-attack". In the past *in vitro *studies of lymphocytes infiltrating colorectal cancer have failed to demonstrate specific anti-tumour activity even though analysis of their receptor usage strongly suggested antigen-specificity [72,73]. These studies did not discriminate tumours according to microsatellite status but one explanation offered for the findings was that the presence of anti-lymphocyte agents, released by the tumour itself, neutralized the activity of tumour infiltrating lymphocytes. The fatty acid synthase (Fas)/Fas-Ligand system has been implicated as a pathway of colorectal cancer counter-attack [74]. Fas-L released by colorectal cancers may bind to Fas receptors on infiltrating lymphocytes and induce their apoptosis. Infiltrating lymphocytes may release Fas-L themselves but colorectal cancers evade the detrimental effects of this by uncoupling their Fas receptors from the intra-cellular machinery that mediates cell death. An immunohistochemical study examining membrane-bound Fas-L expression found that, contrary to expectation, Fas-L expression is not increased in MSS cancers and thus does not explain the less prominent lymphocyte infiltrates seen in these cancers [75]. The authors conclude that some other, as yet unidentified, mechanism of counter-attack must exist in these cancers to account for the reduced infiltrate in MSS colorectal cancer rather than attributing the difference to variable degrees of immunogenicity.

### MSI-H colorectal cancer and apoptosis

Dolcetti and colleagues showed that MSI-H colorectal cancers stained significantly more heavily for markers of apoptotic cell death [76]. Subsequent follow-up analysis of the patients from whom the tumours had been removed showed that increased apoptosis counts correlated well with improved survival [40]. However, Michael-Robinson and co-workers also studied apoptosis in these cancers using TUNEL and though they confirmed the presence of more apoptotic cells, they found no co-localisation between these cells and infiltrating lymphocytes [77]. They concluded therefore that increased T cell infiltrates were not likely to be responsible for more marked tumour cell death. However, it may be argued that the release cytokine mediators of target cell death by infiltrating lymphocytes may render the need for direct co-localisation obsolete. In our opinion as MSI-H colorectal cancer does appear to be infiltrated by an activated cytotoxic T cell population and the tumours show high levels of apoptosis the two are likely to be linked but we acknowledge the need for further clarification.

An alternative explanation for the increased apoptosis observed in MSI-H colorectal cancer has been the theory of "effete malignancy". This concept proposes that the mutator phenotype affects peptides or pathways involved in cell processes fundamental to life. Thus, the accumulation of mutations adversely influences the tumour cells' viability and hence, leads to apoptotic cell death [78]. The anti-apoptotic bcl-2 protein has been noted to be under-expressed in MSI-H colorectal cancers [79] but the pro-apoptotic gene BAX also appears to be a common target for mutation in the MSI pathway [80], an event that may promote clonal expansion and tumourigenesis. These observations confirm the complex role of apoptosis in tumour development and progression.

The apoptosis observed in these cancers, whether induced by effete malignancy or an immune response, is of significance for another reason. The death of tumour cells releases intra-cellular proteins into the tumour micro-environment. This would be a vital step in the promotion of TSA release, recognition and presentation to the host T lymphocytes. It might also release heat shock proteins, upregulated in MSI-H colorectal canecr, which may promote both innate and antigen-specific arms of the immune response. The importance of dendritic cells in immune responses has already been mentioned [49] and immunohistochemical analysis of colorectal cancer has demonstrated the presence of dendritic cells at tumour margins [81]. The presence of this population has not been evaluated in MSI-H cancers specifically but the presence of dendritic cell infiltrates has been associated with reduced tumour progression in colorectal cancer [82].

## Conclusion

Clearly, the mutator phenotype may influence the function of several cellular processes and these may include those that are designed to nullify the host response. Equally it has been suggested that it is the effect of microsatellite instability on other cellular processes, unrelated to tumour immunology, which leads to the survival benefit observed in MSI-H colorectal cancer. Effete malignancy may, for instance, limit a tumour's ability grow and survive [78]. Alternatively processes critical to tumour metastasis may be adversely affected by the mutator phenotype. The down-regulation of VEGF in MSI-H colorectal cancer is a one example of how these tumours may suffer reduced metastatic potential [83]. Certainly many of the biological characteristics of MSI-H, such as diploidy, wild-type p53 expression, activating β-catenin mutations and the CIMP+ phenotype, have all been associated with improved prognosis [84]. However, the effects of all these factors and that of an immune response may be additive, rather than exclusive and they may all contribute to the improvement in outcome.

There is now an accumulation of evidence that promotes the argument that MSI-H colorectal cancer is subject to a marked, possibly antigen-specific, immune response. We acknowledge that this may not be the sole determinant of improved prognosis although it seems likely to be a major influence. We believe that MSI-H may be a natural paradigm of host-tumour interactions in immunogenic colorectal cancers and as such further studies are required to clarify the nature of immune responses in these tumours. The use of heat shock proteins and abnormal peptides, derived from frameshift mutations in coding microsatellites, already hold promise in advancing the field of immunotherapy [85,86]. Ultimately the demonstration of specific anti-tumour activity in cytotoxic lymphocytes from patients with MSI-H colorectal would provide the best evidence in support of such a response. Such studies should enhance our understanding of the immune responses in MSI-H tumours and the tumour immunology of colorectal cancer in general. This may be crucial to the advance of efforts in the field of immunotherapy.

## Competing interests

The author(s) declare that they have no competing interests.

## Authors' contributions

AB searched and evaluated the literature and drafted the manuscript.

SAB and SD revised the manuscript.

All authors read and approved the final manuscript.
